# Antioxidant activities of *Lampaya medicinalis* extracts and their main chemical constituents

**DOI:** 10.1186/1472-6882-14-259

**Published:** 2014-07-21

**Authors:** Glauco Morales, Adrián Paredes

**Affiliations:** 1 Laboratorio de Química Biológica, Instituto Antofagasta (IA), Universidad de Antofagasta, Antofagasta, Chile; 2 Departamento de Química, Facultad de Ciencias Básicas, Universidad de Antofagasta, Antofagasta, Chile

**Keywords:** *Lampaya medicinalis*, Hydroethanolic extract, Antioxidant activity, Flavonoids

## Abstract

**Background:**

*Lampaya medicinalis* Phil. (*Verbenaceae*) is a plant used by Aymara and Quechua ethnic groups from Northern Chile as folk medicine in the treatment and cure of various diseases. The aim of this study was to investigate the *in vitro* antioxidant activity, total phenols content, total flavonoids content, total antioxidant activity, reducing power, brine shrimp cytotoxicity and identify the principal chemical constituents.

**Methods:**

The crude hydroethanolic extract (HEE) and its partitioned fraction: hexane (HF), dichloromethane (DF), ethyl acetate (EAF), n-butanol (BF) and soluble residual aqueous fraction (RWF) were evaluated for their antioxidant activity using different assays namely, DPPH, ABTS, FRAP, β-carotene bleaching assay. The content of total phenolics and total flavonoids were measured by Folin-Ciocalteau and by the AlCl_3_ colorimetric method, respectively. Reducing power was determined by phosphomolybdate and hexacyanoferrate (III) methods. Biotoxicity assays were performed on shrimps of *Artemia salina*. The EAF was fractionated using chromatographic methods.

**Results:**

Considerable amount of phenolic and flavonoid contents were recorded in the hydroethanolic extract (HEE) and its derived fractions. Although HEE and all its derived fractions exhibited good antioxidant activities, the most distinguished radical scavenging potential was observed for ethyl acetate fraction (EAF). EAF showed the higher radical scavenging activity by DPPH (95%) and by ABTS (98%), antioxidant activity by FRAP (158.18 ± 5.79 mg equivalent Trolox/g fraction), β-carotene bleaching assay (86.8%), the highest total phenols content (101.26 ± 1.07 mg GAE/g fraction), the highest total flavonoids content (66.26 ± 3.31 μg quercetin/g fraction). The EAF extract showed an reducing power of 78% and 65% using the phosphomolybdate and hexacyanoferrate (III) assays, respectively.

Four flavonoids, two p-hydroxyacetophenone derivatives and one iridoid were isolated from *Lampaya medicinalis* for the first time.

**Conclusion:**

EtOAc soluble fraction (EAF) shows the strongest antioxidant activity, and it can be attributed to its high content in phenolic and flavonoid compounds. It can be concluded that *L.medicinalis* can be used as an effective natural source of antioxidant, as ethnomedicine and as a commercial basis for the development of nutraceuticals.

## Background

Free radicals are chemical species which contain one or more unpaired electrons in their outer shell. They are highly unstable and cause damage to other molecules by extracting electrons from them in order to attain stability. Free radicals result from an imbalance between the generation of Reactive Oxygen Species (ROS) and the antioxidant protection conferred by enzymatic systems. Since 1950s, free radical reactions are directly involved in aging and progression of several diseases [1,2]. In the past few years, there has been growing interest in the involvement of ROS in several pathological situations. The oxidation induced by ROS can result in cell membrane disintegration, membrane protein damage and DNA mutation, which can further initiate or propagate the development of many diseases, such as cancer, diabetes, liver injury, arteriosclerosis and cardiovascular disease [3]. Although the human body possesses physiological defense mechanisms that reduce the damaging properties of ROS, continuous exposure to various factors and pathological conditions can lead to an increase in the amount of free radicals and cause irreversible oxidative damage. In humans the over-production of ROS can result in tissue injury and has been implicated in disease progression. Free radicals have been associated with several diseases such as inflammation, cardiovascular accidents, cancer, aging-related disorders and atherosclerosis [4].

When there is a lack of materials to quench the excess of reactive free radicals, oxidative stress occurs which can lead to serious diseases. Free radicals then react with various cellular components causing serious damage. So there is a great need for antioxidant supplements to avoid conflict oxidative damage. In the food industry, an antioxidant is defined as a substance that, in low concentrations, is capable of preventing or delaying oxidation of easily oxidizable biomolecules. In mechanistic terms, an antioxidant can be defined as a hydrogen donor or an electron donor. The use of plants as food and medicinal remedies from ancient times is partially attributed to biological efficacy of secondary metabolites that possess antioxidant activities such as phenolic compounds [5]. Medicinal plants are popular remedies used by a majority of the world’s population. The efficacy of medicinal plant in the management of diseases is indubitable. The World Health Organization estimated that 80% of the population of developing countries continues to use traditional medicine in primary medical problems. Over the last few years, investigation of medicinal properties of various plants attracted an increasing interest due their pharmacological activities [6].

Medicinal plants comprise a source of biologically active compounds, which possess different chemical structures and diverse biological activities, and they may, due to the synergistic actions act as powerful antioxidants. Among these compounds, flavonoids and phenolic compounds, phytochemicals with a wide distribution in nature, have been the subject of several studies on their antioxidant activity, which is mainly due to their ability to act as free radical scavengers and/or a metal chelator. There is an increasing interest in the natural antioxidant contained in medicinal plants, which are candidates for the prevention of oxidative damage [7].

Andean High Plateau in Northern Chile, known as “Puna atacameña”, is a particular biotope characterized by varying altitudes of between 3000–4200 meters above sea level, a very low relative humidity, no rains, and cloudless skies during most of the year. Around 5000 people live in this peculiar ecological system and they use medicinal plants as curatives or palliatives of health problems because the plants are recognized as a traditional way to treat ailments and diseases [8].

*Lampaya medicinalis* Phil (*Verbenaceae*) commonly known, as “lampaya” is a small bush, with a height of 80–100 cm that grows in the “Puna atacameña” in Northern Chile. There is little or no information about the chemical composition or medicinal properties of this plant in the scientific literature [9]; however, oral reports from herbal medical practitioners indicate that the infusion of the plant is usually prepared and given for treatment of colds, stomach pain, urinary bladder discomforts, as antitussive, and against rheumatism, arthritis and body joints pain [10-13].

The antioxidant activity of hydroethanolic extract and its partitioned fraction was investigated against several in vitro models. Since free radicals are different chemicals entities, it is essential to test the material against many free radicals to prove its antioxidant activity. This paper reports the evaluation of the antioxidant activities of hydroethanolic extract (HEE) and its partitioned fraction of *Lampaya medicinalis*, using different assays namely, 2,2-diphenyl-1-picrylhydrazyl (DPPH) and 2,2’-Azino bis-(3-ethylbenzothiazoline-6-sulfonic acid (ABTS), β-carotene bleaching assay, Ferric reducing/Antioxidant Power assays (FRAP). The content of total phenolics and total flavonoids were measured by Folin-Ciocalteau and by the AlCl_3_ colorimetric method, respectively.

Reducing power was determined by phosphomolybdate and hexacyanoferrate (III) methods. Biotoxicity assay was performed on shrimps of *Artemia salina*. IC_50_ values obtained were compared with the standards used. In addition, this paper describes chromatographic separation of the EAF fraction from HAE of this plant and the isolation of two p-hydroxyacetophenones, one iridoid and four flavones.

## Methods

### General

Melting point was determined in a Electrothermal 9100 (ThermoFisher Scientific) and no correction were made. The absorption spectra in the infrared were obtained using a FT-IR Nicolet Avatar 330 using KBr pellets. The nuclear magnetic resonance spectra of ^1^H and ^13^C, 1D NMR and 2D NMR were recorded on a Bruker Avance 400 Mhz, spectrometer with TMS as internal standard, using CDCl_3_ and DMSO as solvent. The ultraviolet spectra were recorded on Varian spectrophotometer Model Cary 50. A Thermo Spectronic Genesys20 UV–VIS Spectrometer was used for colorimetric measurement. The Mass Spectra were recorded on Micromass AutoSpec - Ultima NT, spectrometer. Silica gel (200–300 mesh) and silica gel GF_254_ plates were purchased from Merck (Chile). 2,2-diphenyl-1-picrylhydrazyl (DPPH), 2,2’-azino-bis(3-ethylbenzothiozoline-6-sulphonic acid) diammonium salt (ABTS), Folin-Ciocalteu reagent, 6-hydroxy-2,5,7,8-tetramethylchroman-2-carboxylic acid (Trolox), 2,4.6-tri-(2-pyridyl)-s-triazine (TPTZ), β-carotene, trichlroacetic acid, linoleic acid, gallic acid, ascorbic acid, rutin and quercetin were purchased from Sigma-Aldrich (Chile). All other reagents and solvents from local sources were of analytical grade.

### Plant material

Leaves and aerial parts of *Lampaya medicinalis* Phil. were collected at Socaire in Northern Chile (23°36’40 s S; 67°50’33 s W, 3230 m above sea level). The material was identified by Professor Dr. Roberto Rodríguez, Facultad de Ciencias Naturales y Oceanográficas de la Universidad de Concepción, and voucher specimens are kept at the Herbarium (CONC) of Departamento de Botánica de la Universidad de Concepción, Chile.

### Extraction and fractionation procedure

The air-dried leaves of *Lampaya medicinalis* Phil. (4.2 Kg) were chopped and exhaustively extracted with EtOH:H_2_O (1:1, 10 L) during one week at room temperature. The solution was filtered, evaporated in vacuo, and lyophilized to give a dried powder extract (1020 g). This powder (930 g) suspended in 5 l methanol:water (1:9) and partitioned in succession with 5 L of n-hexane, 5 L of dichloromethane, 5 L of ethyl acetate and 5 L of n-butanol. After removal of the solvent in vacuo, the following residues were sequentially obtained: n-hexane-soluble (HF, 20 g), dichloromethane-soluble (DF_,_ 150 g), ethyl-acetate-soluble (EAF, 300 g), butanol-soluble (BF, 250 g), and finally residual water-soluble fraction (RWF, 170 g). A sample (15 g) was scooped out from each fraction, freeze-dried and stored in a refrigerator at 4°C until further use.

### Determination of total antioxidant content

#### Total phenols content

1 mL of the HEE and its derived fractions (HF, DF, EF, BF and RWF) (10 mg/mL) were mixed with 1 mL of Folin-Ciocalteu’s reagent, previously diluted with water 1:10 v/v. After 5 min, 10 mL Na_2_CO_3_ (7%) was added. Mixture was thoroughly mixed with 13 mL of distilled water and incubated at 23°C in the dark. After 90 min, absorbance was recorded at 750 nm. A standard solution of gallic acid serial dilution (25–300 μg/mL in 10% EtOH), was used to prepare a calibration curve. These data allowed to generate the lineal regression; the equation of the direct line was obtained and it was used for the calculation of the experimental concentration of samples. Estimation of phenolic compounds was recorded and expressed as mg of Gallic Acid Equivalents (GAE) per g of dried extract [14]. Data for each extract was recorded in triplicate and expressed as mean value.

#### Total flavonoids content

The total flavonoids content was estimated by a colorimetric procedure using AlCl_3_ and NaOH [15]. Aliquot parts 250 μL of each extract (10 mg/mL, dissolved in respective solvent) was mixed with 1.25 mL of distilled water in a test tube, followed by addition of 75 μL of a 15% w/v NaNO_2_ solution. After 6 min, 150 μL of 10% w/v AlCl_3_ solution was added, and the mixture was allowed to stand for a further 5 min before 0.5 mL of 1 M NaOH was added. The mixture was made up to 2.5 mL with distilled water and mixed well.

The absorbance was measured immediately at 510 nm. A standard solution of quercetin (2.5-250 μg/mL in EtOH) was used to prepare a calibration curve. These data allowed to generate the lineal regression, the equation of the direct line was obtained and it was used for the calculation of the experimental concentration of samples. The results of the samples were expressed as mg quercetin equivalents per g the dry extract.

### Determination of antioxidant activity

#### ABTS assay

HEE, HF, DF, EF, BF and RWF were tested by using the ABTS assay. The assay involve the use of a pre-formed ABTS radical cation (2,2’-azinobis (3-ethylbenzothiozoline-6-sulphonic acid diammonium salt, ABTS^.+^) The assay is based on the discoloration of ABTS by antioxidant compounds, thus reflecting the amount of ABTS radical that are scavenged in relation to that of 6-hydroxy-2, 5, 7, 8-tetramethylchroman-2-carboxylic acid (Trolox) with spectrophotometric analysis [16]. The stock solution of ABTS^.+ ^cation radical was prepared by mixing equal volumes of 7 mM ABTS in H_2_O and 2.45 mM potassium persulfate solution followed by incubation for 12 h at room temperature in the dark to yield a dark - colored solution. Working solution was prepared freshly before each assay by diluting the stock solution with phosphate buffered saline solution (PBS) (pH 7.4) for an absorbance of 0.70 ± 0.02 at 734 nm and equilibrated at 23°C. Free radical scavenging activity was assessed by mixing 300 μL of different fractions (25–250 μg/mL, in respective solvents) with 3 mL of ABTS working solution. The decrease in absorbance was measured exactly 1 min after mixing the solution, the final absorbance was noted up to 6 min. Determinations were repeated three times for each sample solution. The percentage inhibition of absorbance at 734 nm was calculated for each concentration relative to a blank absorbance (methanol), plotted as a function of concentration compound or standard Trolox. Inhibition of ABTS radical scavenging activity was calculated according to the equation AA(%) = [(A_blank_ - A_sample_)/A_blank_] × 100, where A_sample_ is the absorbance of sample or standard, and A_blank_ is the absorbance of the ABTS solution only. All tests were run in triplicate and averaged. The results reported are expressed as their IC_50_ through Graph prism pad software.

### DPPH radical scavenging activity

The free-radical scavenging activity of various fractions, gallic acid and ascorbic acid was measured with the stable radical diphenylpicryhydrazyl (DPPH) in terms of hydrogen donating or radical scavenging activity. DPPH (3.9 mL, 0.075 mM, in methanol) solution was added to 100 μL of different fractions (dissolved in respective solvent), at different concentration (1–250 μg/mL). After storage at room temperature for 30 min, the absorbance was measured at 517 nm against a blank [17]. Inhibition of DPPH radical scavenging activity was calculated according to the equation AA(%) = [(A_blank_ - A_sample_)/A_blank_] × 100, where A_sample_ is the absorbance of sample or standard, and A_blank_ is the absorbance of the DPPH solution only and IC_50_ value was calculated by graph pad prism software. All test were run in triplicate and averaged.

### Ferric reducing/antioxidant power FRAP assay

Ferric reducing/antioxidant power was measured according to the method described by Suarez et al. with some modification [18]. The stocks solutions included 300 mM acetate buffer (3.1 g C_2_H_3_NaO_2_.3H_2_O and 16 mL acetic acid, pH 3.6), 10 mM TPTZ (2,4.6-tripyridyl-s-triazine) solution in 40 mM HCl, and 20 mM FeCl_3_.6H_2_O solution were prepared. The fresh working solution (FRAP reagent) was prepared by mixing 25 mL acetate fuffer, 2.5 mL TPTZ solution, and 2.5 mL FeCl_3_.6H_2_O solution. Plant extract samples (100 μL, 1 mg/mL) and deionized water (300 μL) were mixed with 3 mL of the FRAP reagent and allow to react for 5 min in the dark. Readings of the colored product [ferrous tripyridyltriazine complex] were then taken at 539 nm. The results were expressed as mg of Trolox equivalent/g extract [19].

### β-carotene bleaching method

The inhibition activity of β-carotene oxidation by peroxide radicals of samples was determined as described by Wu et al. [20]. 1 mL of β-carotene solution (0.020% in chloroform) was pipette into a flask containing 20 mg of linoleic acid and 200 mg of Tween 40. After evaporation of chloroform, 100 mL of distilled water was added with vigorous agitation to form an emulsion. 2 mL of this emulsion and 100 μL of sample (50–250 μg/mL) were transferred into a test tube. The mixture was then gently mixed and placed in a water bath at 50°C for 2 h. Absorbance of solution was recorded at 470 nm every 20 min up to 120 min. Quercetin or ascorbic acid can be used as standard antioxidant, positive control. As a negative control was used 50% aqueous methanol in place of the sample. A Tween 20 solution is used as a blank. The results were based upon two different parameters of the antioxidant activity (AA%) and oxidant rate ratio (R_OR_). The rate of β-carotene bleaching (R) was calculated according to equation R = [ln(A_o_/A_t_)]/t, where ln is natural logarithm, A_o_ is Absorbance of the emulsion at time 0, A_t_ is Absorbance at time t, and t is 20, 40, 60, 80, 100 or 120 min. The AA% was calculated according to the following equation: AA % = [R_blank_ - R_sample_/R_blank_] × 100, where R_sample_ and R_blank_ represent the bleaching rates of β-carotene with or without the addition of sample, respectively. The R_OR_ was calculated according to equation R_OR_ = (R_sample_/R_blank_).

### Determination of reducing power

#### Phosphomolybdate method

Reducing power was determined by phosphomolybdate method, using ascorbic acid as standard [21]. Reagent solution included 0.6 M sulfuric acid, 28 mM sodium phosphate and 4 mM ammonium molybdate. Different concentrations (10–200 μg/mL) of sample solutions were prepared from the stock solution. An aliquot of 100 μL of sample solution was mixed with 1 mL of reagent solution. The tubes were capped and incubated in a water bath at 95°C for 90 min. Samples had cooled to room temperature, the absorbance of mixture was measured at 765 nm against the blank. A typical blank contained 1 mL of the reagent solution and the appropriate volume of solvent and incubated under the same conditions. Ascorbic acid was used as standard. Increased Absorbance of the reaction mixture indicated higher total antioxidant capacity. Reducing power was estimated using following formula: Reducing power(%) = [(A_blank_ - A_sample_)/A_blank_] × 100 where A_sample_ is the absorbance of sample, and A_blank_ is the absorbance of blank solution, containing all reagents except the test sample.

#### Hexacyanoferrate (III) assay

The reducing power of all fractions and ascorbic acid were determined based on the method described by Rahman et al. [22]. Different concentrations (10–200 μg/mL) of sample solutions were prepared from the stock solution. 100 μL of sample solution was combined with 2.5 mL of 1% potassium hexacyanoferrate (III). After the mixture was incubated at 50°C for 20 min, 2.5 mL of 10% trichloroacetic acid was added to mixture, which was centrifuged at 3000 rpm for 10 min. Finally, 2.5 mL of the supernatant was mixed with 2.5 mL of distilled water and 0.5 mL of 0.1% FeCl_3_ solution. The absorbance was measured at 700 nm against a blank. The control contained all reagents except the extract fraction while water was used as blank. Ascorbic acid was used as a standard. Higher absorbance indicates higher reducing power. The increase of reducing power by the extract and standard was calculated as follows: Percentage of increase reducing power = [(A_test_ - A_control_)/A_test_] × 100, where A_test_ is absorbance of test solution and A_control_ is absorbance of control [23].

### Biotoxicity assays against *Artemia salina*

The bioactivity of each extract was monitored by the brine shrimp lethality test against *Artemia salina* Leach naupliis according to previously described methods, in three independent 36-h exposure experiments at 22–25°C. *Artemia salina* eggs were incubated at room temperature for 48 h with the help of a light source, in seawater boiled and filtered [24,25]. Ten nauplii were used to test each dose of extract HEE, HF, DF, EAF, BF and RWF (1000, 100 and 10 μg/mL, in triplicates). Survivors were counted with the aid of a magnifying glass. After 36 h of incubation and maintaining the vials under illumination, the deaths at each dose level and controls were determined. The number of dead nauplii were used for calculating the LC_50_ at 95% confidence limit by the Finney Probit analysis program. HgCl_2_ was used as a positive control, methanol was used as solvent, brine shrimp larvae in only sea water were taken as a negative control.

### Statistical analysis

All data were expressed as means ± SD. Analysis of variance was performed by ANOVA procedure. Duncan’s new multiple-range test was used to determine the differences of means, and *p < 0.05* was considered to be statistically significant.

### Isolation and identification of compounds (1–7)

A sample of EAF was separated by successive column chromatography over silica gel using n-hexane and EtOAc gradient with increasing amounts of EtOAc to obtain three crystalline colourless and four crystalline yellow products.

Structure assignments of natural products were done based on their physical and spectroscopic properties: UV, IR, NMR and MS analysis and comparison with data reported in the literature.

### 5–hydroxy -7,4′–dimethoxyflavone

1. Yellow crystal, mp 173–174°C (EtOAc - n-hexane). ^1^H NMR (300 Mz, CDCl_3_) δ 3.88 (3H, s, OMe), 3.90 (3H, s, OMe), 6.58 (1H, s, H-3), 6.37 (1H, d, J=2.2 Hz, H-6), 6.48 (1H, d, J=2.2 Hz, H-8), 7.84 (2H, d, J=9.1 Hz H-2′ and H-6′), 7.02 (2H, d, J=9.1 Hz H-3′ and H-5′), 7.98 (1H, s, OH), 12.10 (H, s, OH-5). ^13^C NMR (75Mz, CDCl_3_, DMSO) δ 163.35 (C-2), 104.27 (C-3), 182.30 (C-4), 157.60 (C-5), 98.20 (C-6), 165.60 (C-7), 92.90 (C-8) 152.60 (C-9), 105.00 (C-10), 122.59 (C-1′), 126.36 (C-2′), 114.48 (C-3′), 161.63 (C-4′), 114.48 (C-5′), 126.36 (C-6′), 56.10 (OMe, C-4′), 55.00 (OMe, C-7).

### 5–hydroxy–7,3′,4′-trimethoxyflavone

2. Yellow needles, mp 163–165°C (EtOAc–n-hexane). ^1^H NMR (300 Mz, CDCl_3_) δ 3.82 (3H, s, OMe), 3.85 (3H, s, OMe), 3.89 (3H, s, OMe), 6.50 (1H, s, H-3), 6.29 (1H, d, J=2.3 Hz, H-6), 6.41 (1H, d, J=2.3 Hz, H-8), 7.25 (1H, d, J=2.2 Hz H-2′), 6.91 (2H, d, J=8.6 Hz H-5′), 7.45 (1H, dd, J=8.6, 2.2 Hz H-6′), 12.10 (H, s, OH-5). ^13^C NMR (75Mz, CDCl_3_) δ 163.80 (C-2), 103.07 (C-3), 179.14 (C-4), 163.30 (C-5), 98.20 (C-6), 167.60 (C-7), 92.90 (C-8), 156.60 (C-9), 106.00 (C-10), 122.99 (C-1′), 114.90 (C-2′), 147.48 (C-3′), 149.00 (C-4′), 114.90 (C-5′), 121.00 (C-6′), 56.50 (OMe, C-4′), 56.20 (OMe, C-3′), 55.90 (OMe, C-7).

### 5,4′-dihydroxy–7,3′-dimethoxyflavone

3. Yellow needles, velutin, mp 216–218°C (EtOAc - n-hexane). ^1^H NMR (300 Mz, CDCl_3_) δ 3.81 (3H, s, OMe), 3.93 (3H, s, OMe), 6.42 (1H, s, H-3), 6.30 (1H, d, J=2.3 Hz, H-6), 6.51 (1H, d, J=2.3 Hz, H-8), 7.25 (1H, d, J=2.2 Hz H-2′), 6.97 (2H, d, J=8.6 Hz H-5′), 7.43 (1H, dd, J=8.6, 2.2 Hz H-6′), 12.72 (H, s, OH-5). ^13^C NMR (75Mz, CDCl_3_) δ 163.92 (C-2), 103.23 (C-3), 180.62 (C-4), 161.05 (C-5), 96.30(C-6), 165.15 (C-7), 92.70 (C-8) 155.90 (C-9), 106.50 (C-10), 123.59 (C-1′), 114.90 (C-2′), 147.48 (C-3′), 145.10 (C-4′), 119.00 (C-5′), 121.60 (C-6′), 55.75 (OMe, C-4′), 55.90 (OMe, C-7),

### 5,4′–dihydroxy–6,7,3′-trimethoxyflavone

4. Yellow needles, cirsilineol, mp 204–206°C (EtOAc–n-hexane). ^1^H NMR (300 Mz, DMSO) δ 3.75 (3H, s, OMe), 3.91 (3H, s, OMe), 3.93 (3H, s, OMe), 6.86 (1H, s, H-3), 6.94 (1H, s, H-8), 7.59 (1H, d, J=2.2 Hz H–2′), 6.95 (2H, d, J=8.6 Hz H-5′), 7.58 (1H, dd, J=8.6, 2.2 Hz H-6′), 12.95 (H, s, OH-5). ^13^C NMR (75Mz, CDCl_3_) δ 163.98 (C-2), 103.23 (C-3), 182.62 (C-4), 162.65 (C-5), 131.90 (C-6), 158.65 (C-7), 91.68 (C-8), 152.50 (C-9), 106.50 (C-10), 121.39 (C-1′), 103.10 (C-2′), 148.10 (C-3′), 150.78 (C-4′), 120.50 (C-5′), 118.70 (C-6′), 56.40 (OMe, C-4′), 55.99 (OMe, C-7), 60.00 (OMe, C-6).

### p-hydroxyacetophenone

5. Colorless crystal, mp 109–111°C. ^1^H NMR (300 Mz, CDCl_3_) δ 2.57 (3H, s, COMe), 6.54 (1H, s, OH) , 6.92 (2H, d, J=8.8 Hz H-3 and H-5), 7.91 (2H, d, J=8.8 Hz H-2 and H-6). ^13^C NMR (75Mz, CDCl_3_) δ 26.25 (Me), 115.53 (C-3 and C-5), 129.66 (C-1), 131.19 (C-2 and C-6), 161.27 (C-4), 198.50 (CO).

### 4–O–β–D–glucopyranosyl acetophenone

6. Colorless crystal, picein, mp 195°C. ^1^H NMR (300 Mz, DMSO) δ 2.38 (3H, s, COMe), 3.20 (1H, m, H-4′), 3.30 (1H, m, H-2′), 3.32 (1H, m, H-3′), 3.70 (1H, m, H-5′), 4.41 (1H, d J =11.0 Hz H-6′), 4.17 (1H, dd J=11.0 and 7.3 Hz H-6′), 5.07 (H, d, J=9.0 Hz H-1′). ^13^C NMR (75Mz, CDCl_3_) δ 26.25 (Me), 116.07 (C-3 and C-5), 130.98 (C-1), 130.23 (C-2 and C-6), 160.92 C-4), 196.45 (CO), 63.30 (C-6′), 70.15 (C-4′), 73.23 (C-2′), 74.02, (C-5′), 76.43, (C-3′), 99.62 (C-1′).

### Genipin

7. Colorless crystal, mp 120 – 122°C (Et_2_O). ^1^H NMR (300 Mz, CDCl_3_) δ 2.08 (1H, m, H-6), 2.55 (1H, m, H-9), 2.90 (1H, m, H-6), 3.22 (1H, m, H-5), 3.74 (3H,s, COOMe), 4.33 (2H, d, J=8.6 Hz H-10), 4.83 (1H, d, J=8.6 Hz H-1) 5.88 (1H, H-7), 7.53 (1H, H-3). ^13^C NMR (75Mz, CDCl_3_) 168.17 (COOMe), 152.74 (C-3), 142.26 (C-8), 130.13 (C-7), 110.96 (C-4), 95.33 (C-1), 61.53 (C-10), 51.50 (Me), 48.38 (C-9), 39.24 (C-6), 36.22 (C-5).

## Results and discussion

### Determination of total antioxidant content

#### Total phenols assay and total flavonoids assay

Polyphenols are the most abundant antioxidants in the plant kingdom and it is likely that the antioxidant capacity of extract/fraction might be due to these compounds. Table 1 shows the quantity of total phenolics and total flavonoids content. Total phenols content varied from 101.26 ± 1.07 to 27.64 ± 0.39 mg Gallic acid equivalent/g extract/fraction. Maximum quantity of total phenol content was observed in EAF, 101.26 ± 1.07 mg GAE/g fraction, with a significant difference with the rest of the fractions, followed by HEE and RWF with 49.18 ± 0.86 mg GAE/g fraction and 48.41 ± 0.91 mg GAE/g fraction, respectively. BF and DF showed the lowest content of phenols with 36.10 ± 0.37 mg GAE/g fraction and 27.64 ± 0.39 mg GAE /g fraction, respectively.

**Table 1:** **Total phenolics, flavonoids contents and antioxidant activities of diferent fractions from HEE of *L. medicinalis***

**Extract**	**Total phenolics**^**a**^	**Total flavonoids**^**b**^	**IC**_**50 **_**ABTS**^**c**^	**IC**_**50 **_**DPPH**^**c**^	**FRAP**^**d**^	**IC**_**50 **_**β-carotene**^**c**^	**IC**_**50 **_**molybdate**^**c**^	**IC**_**50 **_**Hexacyanoferrate (III)**^**c**^
**HEE**	49.18 ± 0.86^e^	60.04 ± 6.13^e^	11.59 ± 1.21^e^	13.79 ± 0.76 ^e, f^	38.29 ± 1.79^e^	189.07 ± 3.13	48.31 ± 3.13	61.56 ± 5.13
**HF**	----	11.14 ± 2.24	56.32 ± 1.70	27.17 ± 0.64	----	-----	-----	-----
**DF**	27.64 ± 0.39	29.29 ± 3.58^f, g^	19.74 ± 1.17^f^	20.35 ± 0.98	56.90 ± 4.57	252.60 ± 4.87	26.93 ± 2.09	110.5 ± 3.13
**EAF**	101.26 ± 1.07	66.26 ± 3.3^e^	5.16 ± 1,09	9.94 ± 0.18	158.18 ± 5.79	65.41 ± 3.56	41.86 ± 1.56^e^	40.23 ± 3.89
**BF**	36.10 ± 0.37	31.64 ± 2.99^f^	14.12 ± 1.45^e^	15.09 ± 1.05^e^	109.36 ± 5.90	117.69 ± 2.98	37.33 ± 2.19^f^	141.62 ± 4.23
**RWF**	48.41 ± 0.9^e^	23.39 ± 1.77^g^	22.27 ± 2.10^f^	12.49 ± 0.97^f^	31.79 ± 4.56^e^	370.96 ± 3.67	40.47 ± 1.86^e,f^	162.45 ± 5.11

HEE (crude hydroethanolic extract), HF (hexane fraction), DF (dichlorometane fraction), EAF (ethyl acetate fraction), BF (n-butanol fraction), RWF (residual water fraction).

All values were expressed as Means ± SD (n = 3).

^a^expressed in mg Gallic acid equivalent/g extract.

^b^expressed in μg Quercetin/mg extract.

^c^expressed in μg/mL extract.

^d^expressed in mg Trolox equivalent/g.

^e, f, g^extract in the same column followed by different letter are not significantly different (*p < 0.05*).

The total flavonoid content varied from 66.26 ± 3.31 to 11.14 ± 2.24 μg quercetin equivalent/g extract/fraction. Maximum quantity of total flavonoid content was observed in EAF 66.26 ± 3.31 μg quercetin/g fraction, followed by HEE with 60.04 ± 6.13 μg quercetin/g fraction. BF, DF and RWF showed total flavonoid content with 31.64 ± 2.99 μg quercetin/g fraction, 29.29 ± 3.58 μg quercetin/g fraction and 23.39 ± 1.77 μg quercetin/g fraction, respectively. The lowest quantity was observed in HF with 11.14 ± 2.24 μg quercetin/g fraction. It was noted that EAF had the highest total phenol and total flavonoid contents among all fractions.

### Determination of antioxidant activity

Antioxidant properties of different plant extracts can be evaluated using various *in vitro* assays. Antioxidant assays in biological system can assess lipid peroxidation or can measure free radical scavenging ability. For measuring free radical scavenging ability, methods are grouped into two groups, according to the chemical reaction involved: hydrogen radical transfer and single electron transfer. Single electron transfer based-methods detect the ability of an antioxidant to transfer one electron to reduce any compound, including metals and radicals. Methods based on this principle include a substrate oxidant that abstracts an electron from the antioxidant, causing color changes of the substrate. The degree of color change is proportional to the antioxidant concentrations.

### ABTS assay

**Figure 1:**
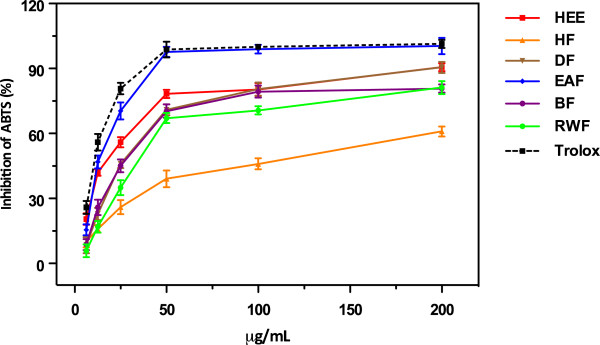
**ABTS scavenging activities of crude HEE and its derived fraction of *L. medicinalis.***

ABTS radical scavenging activity involves a more drastic radical, chemically produced and is often used for screening complex antioxidant mixtures such as plant extracts, beverages and biological fluids. The actual version of this assay, a stable ABTS radical cation which has a blue-green chromophore absorption, was produced by oxidation of ABTS with potassium persulfate. Antioxidant activity of the natural products is determined by the discoloration of the ABTS, by measuring the reduction of the absorbance at 734 nm. The extension of discoloration, expressed as a percentage inhibition of ABTS^.+^, is determined as a function of the concentration and it is calibrated against Trolox as the reference standard. The results of ABTS scavenging activity of hydroethanolic extract (HEE) and its partitioned fraction of *Lampaya medicinalis*, are summarized in Table 1 (IC_50_ values). Overall, the IC_50_ of ABTS free radical scavenging capacity of six extracts was found between 5.16 ± 1.09 to 56.32 ± 1.70 μg/mL. EAF, HEE, and BF showed comparatively strong ABTS free radical scavenging capacity. Similarly, DF and RWF showed moderate activity. Figure 1 shows the dose–response curves of ABTS radical activities of extracts from *Lampaya medicinalis*. It was found that the radical-scavenging activities of all extracts increased with the concentration. All test samples exhibited potent scavenging activities in a concentration dependent manner. At a concentration of 50 μg/mL, scavenging activities of EAF reached a plateau of 98% while, at same concentration, scavenging effects of HEE, DF, BF, RWF and HF were 78%, 71%, 70%, 67%, and 39%, respectively. While at the same concentration the activity of Trolox was 99%. The antioxidant activity of HEE and its fractions and standard Trolox increased in the following order HF < RWF < BF < DF < HEE < EAF ≈ Trolox.

### DPPH radical scavenging capacity

**Figure 2:**
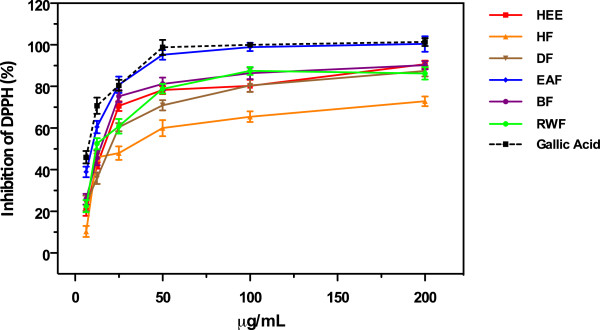
**DPPH scavenging activities of crude HEE and its derived fraction of *L. medicinalis.***

2,2-diphenyl-1-picrylhydrazyl (DPPH), in solution, is a stable free radical, which has been widely accepted as a tool for estimating free radical scavenging activities of antioxidants. One of the reasons is that this method is simple and highly sensitive. This assay is based on the theory that a hydrogen donor is an antioxidant. DPPH is one of the few stable and commercially available organic nitrogen radicals. The antioxidant effect is proportional to the disappearance of DPPH˙ in test sample. Various methods of monitoring the amount of DPPH˙ in the antioxidant test system have been reported, however monitoring with UV spectrometer has become the most widely used. DPPH˙ accepts an electron or hydrogen radical to become a stable diamagnetic molecule. This reaction is stoichiometric with respect to the number of hydrogen atoms absorbed, or to the number of electrons captured. The reduction capability of DPPH radical is determined by the decrease in absorbance at 517 nm induced by antioxidants. Then, color changing from purple to yellow is the consequence of the reducing ability of antioxidant toward DPPH stable free radical. The higher the discoloration of the DPPH methanol solution, the lower the absorbance of the reaction mixture, indicating thereby significant free radical scavenging capacity. The results have been reported as IC_50_, which is the amount of antioxidant necessary to decrease the initial DPPH˙ by 50% expressed in μg/mL. The results of DPPH scavenging activity of hydroethanolic extract (HEE) and its partitioned fraction of *Lampaya medicinalis*, are summarized in Table 1 (IC_50_ values). Overall, the IC_50_ of DPPH free radical scavenging capacity of six samples were found between 9.94 ± 0.18 to 27.17 ± 0.64 μg/mL. EAF, RWF, HEE and BF, showed comparatively strong DPPH free radical scavenging capacity. Similarly, DF and HF showed moderate activity. Figure 2 shows the dose–response curves of DPPH radical activities of extracts from *Lampaya medicinalis*. It was found that all test samples exhibited potent scavenging activities in a concentration dependent manner. At a concentration of 50 μg/mL, the scavenging activities of EAF reached a plateau of 95% while, at same concentration, the scavenging effects of BF, RWF, HEE, DF and HF were 81% , 79% , 74%, 71% and 60%, respectively.

### FRAP assay

Ferric reducing antioxidant power assay is based on the ability of an antioxidant to reduce Fe^+3^ to Fe^+2^ ions in the presence of TPTZ (2,4,6-tris-(2-pyridyl)-s-triazine), forming an intense blue Fe-TPTZ complex with an absorbance maximum at 593 nm. Decreased absorbance is proportional to the antioxidant content. The FRAP assay results of the HEE and its fractions expressed as mg equivalent of Trolox/g fraction are shown in Table 1. EAF showed the highest reducing power with 158.18 ± 5.79 mg Trolox equivalent, followed by BF with 109.36 ± 5.90 mg Trolox equivalent. FRAP values varied from 158.18 ± 5.79 to 31.78 ± 4.56 mg Trolox equivalent. The ferric reducing antioxidant power of HEE and its fractions and Trolox increased in the following order RWF < HEE < DF < BF < EAF ≈ Trolox.

### β-carotene bleaching method

**Figure 3:**
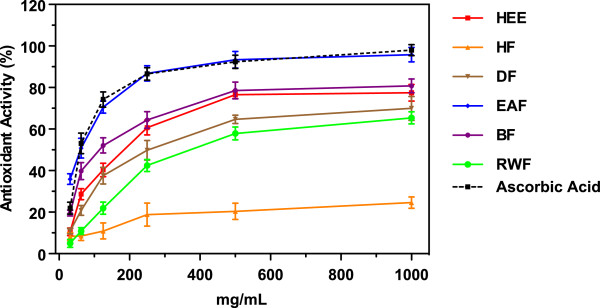
**Antioxidant activity of *L. medicinalis *** **extracts measure by β-carotene bleaching method.**

It is widely know that β-carotene reacts with the peroxyl radical to produce β-carotene epoxides acting as a radical scavenger or antioxidant. The linoleic acid forms a peroxyl radical in the presence of ROS and O_2_. In this antioxidant assay a mixture of β-carotene and linoleic acid is used. The peroxyl radical reacts with β-carotene, subsequently, the amount of β-carotene reduces in a testing solution, resulting in the rapid disappearance of color. If an antioxidant is present in a testing solution, it reacts competitively with peroxyl radical and color is maintained for a longer period. Therefore, antioxidant activities are monitored by bleaching the color of a test solution at 470 nm, which is the typical absorbance by β-carotene. The IC_50_ values of β-carotene bleaching assay of *Lampaya medicinalis* extract and various fractions is summarized in Table 1. Maximum activity was observed by EAF and lowest by RWF with IC_50_ values of 65.41 ± 3.56 μg/mL and 370.96 ± 3.67 μg/mL respectively. The antioxidant activity (%) of HEE and its fractions and ascorbic acid increased with an increasing concentration of the sample. The antioxidant activity of HEE and its fractions and ascorbic acid increased in the following order HF < RWF < DF < HEE < BF < EAF ≈ ascorbic acid. At a concentration of 250 μg/mL, EAF and ascorbic acid exhibited 86.78 ± 3.65% and 86.56 ± 2.98% as antioxidant activity, respectively. Figure 3 shows the dose–response curves of β-carotene bleaching assay of *Lampaya medicinalis* extract and various fractions.

### Reducing power

#### Phosphomolybdate assay

**Figure 4:**
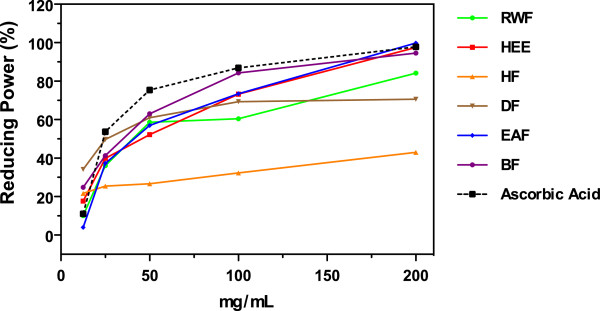
**Reducing power of *L. medicinalis *** **extracts measured by phosphomolybdate method.**

The total antioxidant potential of *Lampaya medicinalis* extracts and various fractions was estimated from their ability to reduce de Mo (VI) to Mo (V) and resulting in formation of green phosphate/Mo (V) complex with maximum absorption at 695 nm. Increased Absorbance of the reaction mixture indicated increased total antioxidant capacity. In the present assay all the different fractions showed good total antioxidant index, which was concentration-dependent. The results of reducing power of hydroethanolic extract (HEE) and its partitioned fraction are summarized in Table 1 (IC_50_ values). Overall, the IC_50_ of five samples were found between 26.93 ± 2.09 to 48.31 ± 3.13 μg/mL. Figure 4 shows the dose–response curve of total antioxidant capacity of extracts from *Lampaya medicinalis*. It was found that all test samples exhibited potent activities in a concentration dependent manner. At a concentration of 100 μg/mL, the antioxidant activity of HEE, its fractions and ascorbic acid increased in the following order HF < RWF < DF < HEE < EAF < BF ≈ Ascorbic acid.

#### Hexacyanoferrate (III) assay

Reduction power, widely used in evaluating antioxidant activity of plants, was determined by the potassium hexacyanoferrate (III) reduction method. In this assay, the presence of reductans in the antioxidant sample causes the reduction of the Fe^+3^/Hexacyanoferrate (III) complex to Fe^+2^/Hexacyanoferrate (III) complex, so the reducing power of sample can be monitored by measuring the formation Perl’s Prussian Blue complex at 700 nm. Increased absorbance indicated increase antioxidant capacity.

**Figure 5:**
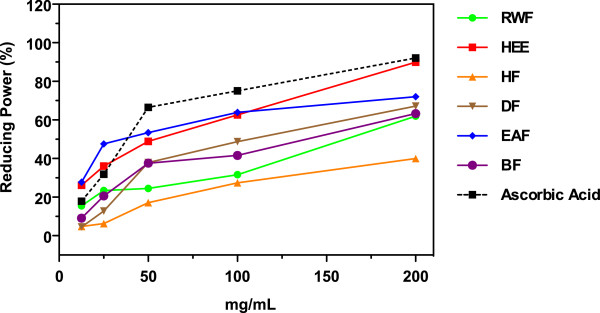
**Reducing power of *L. medicinalis *** **extracts measured by hexacyanoferrate (III) method.**

The IC_50_ values of reducing power assay of *Lampaya medicinalis* extract and various fractions are summarized in Table 1. Maximum activity was observed by EAF and lowest by RWF with IC_50_ values of 40.23 ± 3.89 μg/mL and 162.45 ± 5.11 μg/mL respectively. The reducing activity (%) of HEE and its fractions and ascorbic acid increased with an increasing concentration of the sample in the following order HF < RWF < DF < HEE < BF < EAF ≈ ascorbic acid, as shown in Figure 5. At a concentration of 100 μg/mL, the reducing effects of ascorbic acid, EAF, HEE, DF, BF RWF, and HF were 75%, 64%, 63%, 49%, 42% and 32%, 27%, respectively.

### Biotoxicity assay against *Artemia salina*

**Figure 6:**
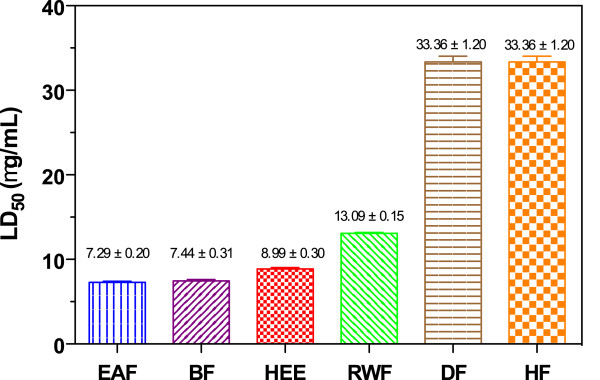
***Biotoxicity assays of L. medicinalis *** **extracts *against Artemia salina.***

A general bioassay that seems capable to detecting a wide range of biological properties of crude extracts is the brine shrimp lethality test. The technique is simple, inexpensive and use a small amount of test material, and it has been used to evaluate plants for potential pharmacological activity. In bioactivity evaluation by brine shrimp bioassay, cytotoxic activity is considered weak when the LC_50_ is between 500–1000 μg/mL, moderate the LC_50_ is between 100–500 μg/mL, and strong when the LC_50_ is between 0–100 μg/mL [26]. The degree of lethality was found to be directly proportional to the concentration of samples. The results of brine shrimp cytotoxicity studies are expressed in Figure 6. 100% of the larvae of *Artemia* were alive after 36 h of experiment in the negative control. All larvae in HgCl_2_ control died at dose 3 μg/mL. Test samples showed LC_50_ in the range of 33.36 ± 1.20 - 7.29 ± 0.20 μg/mL, indicating a strong cytotoxicity activity. Among all tested fractions, EAF (7.29 ± 0.20 μg/mL), BF (7.44 ± 0.31 μg/mL) and HEE (8.99 ± 0.30 μg/mL) showed comparatively higher cytotoxic activity. While less bioactive fractions were HF and DF, with LC_50_ 33.36 ± 1.20 μg/mL. This result indicates that all fractions of *Lampaya medicinalis* may contain chemical compounds with pharmacological properties.

### Isolation and characterization of compounds 1–7

**Figure 7:**
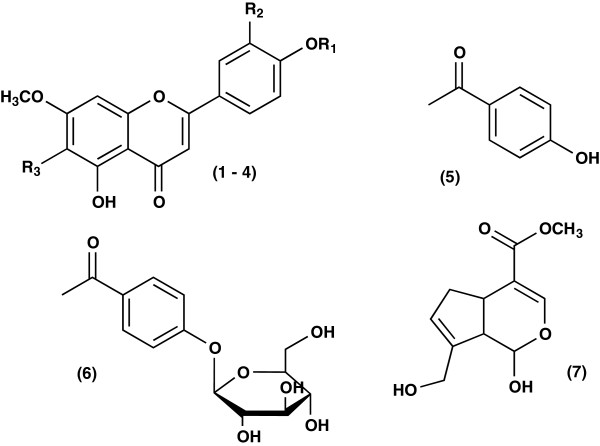
**Structures of compounds *1*–*7 isolated from L. medicinalis.***

This is the first report of the presence of seven compounds in *Lampaya medicinalis*. The structures of the compounds were based on detailed analysis of UV, IR, 1DNMR, 2DNMR, EM spectral evidence and by comparing data with the literature values. Chromatographic fractionation of EAF led to identification of four compounds belonging to the flavonoid class (1–4), two p-hydroxyacetophenone derivatives (5–6) and one iridoid (7) (Figure 7). The chemical shifts of the signals assignable to the C-2, C-3 and C-4 of the ring C in the ^13^C NMR spectra of compounds 1–4 strongly indicates that these flavonoids belong to the class of flavones. Indeed, these signals are characteristic values and within known ranges for C-2 ~ δ 164, C-3 ~ δ 103 and C-4 ~ δ 180, values according to the system double bond conjugated with the carbonyl in C-4 in the ring C [27]. In the ^1^H NMR spectra of compounds 1–4 the signal at δ ~ 12.3 is assignable to OH group attached at C-5 strongly chelated to CO at C-4. On the basis of physicochemical properties, spectral data (UV, IR, 1D NMR, 2D NMR, EI-MS). Compound 1 have oxygenation patterns as apigenin 5,7,4′, Compounds 2 and 3 have oxygenation patterns as luteolin 5,7,3′,4′ and compound 4 is a 6-luteolin substituted and by comparing the data with the literature values the structures were identified to be 5-hydroxy-7,4′-dimethoxyflavone 1 [28], 5-hydroxy-7,3′,4′-trimethoxyflavone 2 [29,30], 5,4′-dihydroxy-7, 3′-dimethoxyflavone, velutin 3 [30-32], 5,4′-dihydroxy-6,7,3′-trimethoxyflavone, cirsilineol 4 [33,34]. 5,4′-dihydroxy-7,3′-dimethoxyflavone, velutin 3 was isolated earlier from several plants [32,35,36].

This compound possesses a strong anti-inflammatory effect inhibiting the expression of proinflammatory cytokines tumor necrosis factor TNF-α and interleukin IL-6 in very low micromole levels by inhibiting nuclear factor NF-κB activation and p38 and JNK phosphorylation in macrophages [37]. 5,4′-dihydroxy-6,7,3′-trimethoxyflavone, cirsilineol 4 was isolated and identified earlier from several plants [38-40]. This compound is interesting for its pronounced antibacterial and antitumor activity [41,42].

Compounds 5 and 6 are p-hydroxyacetophenone and its glucoside 4-O-β-D-glucopyranosylacetophenone, picein, respectively [43-47]. Compound 7 Genipin, C_11_H_14_O_5_, is an aglycone derived from the iridoid glycoside geniposide, which is found in the fruit of *Gardenia jasminoides*[48,49]. Genipin is a hydrolytic product of geniposide. The structure of genipin was discovered in the 1960’s. Because it is a naturally occurring biodegradable molecule with low cytotoxicity, genipin has recently been investigated as a crosslinking material in many biological applications. Recent explorations into the use of genipin crosslinking gelatin for use as a bioadhesive, wound dressing, and as bone substitute, have shown it to have potential as a new and safe crosslinking agent. In the area forensic science, genipin is being examined as a new way of developing latent fingerpring on paper products. Because it is an environmentally friendly natural product it shows great potential over the current reagent being used, ninhydrin. It is reported that genipin has many medicinal effects, such as anti-inflammatory [50], anticancer, anthitrombotic, antibacterial, gastritis curative diabetes curative [51].

## Conclusions

The results obtained in this study demonstrate that the hydroethanolic extract of *Lampaya medicinalis* has strong antioxidant activity in the all methods tested. Its EtOAc soluble fraction (EAF) shows the strongest antioxidants activity and it can be attributed to its high content in phenolic and flavonoid compounds. It can be concluded that *L.medicinalis* can be used as an effective natural source of antioxidant, as ethnomedicine and as a commercial basis for the development of nutraceuticals.

## Competing interests

The authors declare that they have no competing interests.

## Authors’ contributions

GM and AP performed all experiments and read and approved the final manuscript.

## Pre-publication history

The pre-publication history for this paper can be accessed here:

http://www.biomedcentral.com/1472-6882/14/259/prepub
